# Drug Expenditure, Price, and Utilization in US Medicaid: A Trend Analysis for New Multiple Myeloma Medications from 2016 to 2022

**DOI:** 10.3390/healthcare11162265

**Published:** 2023-08-11

**Authors:** Marwan Alrasheed, Abdulrahman Alsuhibani, Bander Balkhi, Jeff Jianfei Guo

**Affiliations:** 1 Department of Clinical Pharmacy, College of Pharmacy, King Saud University, P.O. Box 2454, Riyadh 11451, Saudi Arabia; bbalkhi@ksu.edu.sa; 2 James L. Winkle College of Pharmacy, University of Cincinnati Academic Health Center, Cincinnati, OH 45267, USA; alsuhian@mail.uc.edu (A.A.); guoje@ucmail.uc.edu (J.J.G.); 3 Department of Pharmacy Practice, Unaizah College of Pharmacy, Qassim University, Unaizah 56434, Saudi Arabia

**Keywords:** multiple myeloma, drug expenditure, drug utilization, Medicaid

## Abstract

Introduction: Multiple myeloma (MM) is the most common plasma cell tumor type. In late 2015, the FDA approved three new medications for MM. These medications were ixazomib, daratumumab, and elotuzumab. However, their utilization, reimbursement, and price in the Medicaid program have not been analyzed before. Methods: A retrospective drug utilization study using the national Medicaid pharmacy claims data from 2016 to 2022 in the US. The primary metrics of analysis were utilization (number of prescriptions), reimbursement (total spending), and price (reimbursement per prescription). Results: The overall Medicaid utilization of MM medications increased from 1671 prescriptions in 2016 to 34,583 prescriptions in 2022 (1970% increase). Moreover, the overall Medicaid reimbursement for the new MM medications increased from USD 9,250,000 in 2016 to over USD 214,449,000 in 2022 (2218% increase). Daratumumab had much higher utilization, reimbursement, and market shares than its competitors. Ixazomib was the most expensive medication compared to daratumumab and elotuzumab. Conclusion: The results of this study demonstrate that CMS utilization and spending on MM medications have significantly grown since 2016. Daratumumab has by far the highest utilization, spending, and market share. The utilization of and spending on specific pharmaceuticals are clearly impacted by policy and clinical guideline recommendations.

## 1. Introduction

Multiple myeloma (MM) is a rare but significant hematological malignancy characterized by uncontrolled plasma cell proliferation; this can lead to the buildup of abnormal plasma cells in the bone marrow, which can damage bones, leading to various complications and adverse health outcomes. It poses a significant clinical and economic burden, impacting patients’ quality of life and placing pressure on healthcare systems [1]. Patients with MM experience physical and emotional symptoms, including bone pain, anemia, renal dysfunction, immunodeficiency, fatigue, and reduced quality of life [2]. Furthermore, the frequent medical visits, laboratory tests, imaging studies, and hospitalizations further amplify the overall burden on patients and their families [3].

Multiple myeloma has shown a marked increase in its prevalence. This is strikingly evident in the United States, where the MM cases in 2020 amounted to 144,922, with projections suggesting a rise to approximately 162,339 cases by 2025, according to [4]. This rise can be attributed to various factors, including the aging population, longer life expectancy, and improved diagnostic capabilities. MM represents a non-trivial 1% of all cancers and 10% of all hematological malignancies. Over 32,000 new MM cases are identified annually in the US. This serious situation underscores the urgent necessity to confront the clinical and economic impacts of MM [5]. It highlights the need for effective strategies to alleviate this disease’s growing burden. It is important to note that while the rising prevalence of MM appears alarming, it also reflects advancements in medical knowledge and the ability to diagnose the disease accurately. Early detection enables the prompt initiation of treatment, which can improve patient outcomes and potentially increase survival rates. Moreover, the increasing awareness of MM and the associated symptoms among healthcare professionals and the general public contributes to more cases being identified and reported.

The management of MM necessitates comprehensive treatment regimens, including chemotherapy, immunotherapy, targeted therapy, and stem cell transplantation. The fundamental management objective is to curb disease progression and improve survival rates. However, achieving this goal can be challenging due to disease heterogeneity, potential treatment resistance, and relapse [6,7]. The intensive management of patients with MM requires the use of multiple medications from different classes to control the disease and reduce its progression. These medications are either immunomodulatory agents such as lenalidomide, corticosteroids such as dexamethasone, monoclonal antibodies such as daratumumab and elotuzumab, or proteasome inhibitors such as ixazomib and bortezomib. Advancements in MM medications have positively impacted treatment practices by offering better efficacy and survival outcomes [8]. These medications increase the likelihood of remission, prolong progression-free survival, and enhance patients’ quality of life. Nonetheless, these new medications often come with higher prices, which directly influence the expenditure associated with MM treatment, particularly within Medicaid, where financial resources may be limited [9].

With the rising prevalence of MM, longer treatment durations, and the utilization of a combination of multiple medications, the overall expenditure for MM medication is expected to increase. A study examining the economic burden of MM found that patients with relapsed or refractory MM (RRMM) had considerably higher healthcare costs compared to non-MM patients. RRMM patients had 4.9 times higher total healthcare costs, with pharmacy costs accounting for the majority (67.3%) of the total costs [10]. These findings align with a study of a real-world administrative claims database, which showed that pharmacy and hospital inpatient utilization together constituted two thirds of the total direct healthcare costs for MM patients [11].

Given the escalating costs of medication, drug expenditures have become a critical concern within healthcare systems. The affordability and accessibility of medications directly impact patient care. In the United States, the Medicaid program plays an instrumental role in providing healthcare coverage to low-income individuals, including vulnerable populations. However, the rising cost of pharmaceuticals poses significant challenges in ensuring the availability of essential medications while maintaining sustainable budgets. Overall, understanding the factors driving the rising expenditure for MM medications within the Medicaid program is crucial in assessing the economic implications, ensuring affordability, and improving the accessibility of these drugs for Medicaid beneficiaries. This study aims to conduct a trend analysis to illuminate the patterns of drug expenditure, pricing, and utilization for new multiple myeloma medications. The findings of this study provide valuable insights for policymakers and healthcare providers in developing evidence-based strategies to address the rising expenditure of MM medications within the Medicaid program and similar healthcare programs. These insights can guide the development of strategies to optimize resource allocation, enhance patient care, and navigate the financial implications within Medicaid and similar healthcare programs. By addressing the economic challenges associated with MM treatment, healthcare systems can work towards providing better access to essential medications and improving the overall management of this complex disease.

## 2. Materials and Methods

This was a retrospective drug utilization study using the database of a national Medicaid pharmacy claims database provided by the Centers for Medicare and Medicaid Services (CMS). The study focused on three MM medications approved by the FDA in 2015. The evaluated drugs were the proteasome inhibitor ixazomib (Ninlaro^®^), a monoclonal antibody that attaches to the SLAMF7 protein, elotuzumab (Empliciti^®^), and a monoclonal antibody that attaches to the CD38 protein, daratumumab (Darzalex^®^). Data collection encompassed the period from the drugs’ market entry in late 2015 until the end of 2022, allowing for a comprehensive assessment of their utilization and reimbursement patterns. The study analyzed data on a quarterly basis, providing a granular view of the utilization and spending trends over time.

### 2.1. Statistical Analyses

The primary categories of analysis included the following.

1. Utilization: The number of prescriptions for each MM medication was recorded and analyzed. Utilization was calculated by aggregating the number of prescriptions for each drug per quarter, and yearly utilization was determined by summing the four quarters of each year. This metric reflects the frequency of drug utilization and provides insights into prescribing patterns and trends.
2. Reimbursement: The total spending by Medicaid on each MM medication was assessed. This category quantifies the financial burden associated with these drugs and offers insights into the healthcare system’s expenditure for MM treatment. Reimbursement was calculated for each drug quarterly and then yearly. The currency used for the study was US dollars.
3. Price: The price of each MM medication was calculated by dividing the total reimbursement by the number of prescriptions, yielding the reimbursement amount per prescription. This metric serves as a proxy for the medication’s price and contributes to understanding the pricing and affordability of the studied drugs.

All statistical analysis was performed using the SAS software package for Windows (Version 9.4; SAS Institute, Inc., Cary, NC, USA) and Microsoft Excel 2019 (version 1808).

Trend analysis was performed in Microsoft Excel by visualizing the data for each parameter over time using a line chart with markers. The line for each parameter either increased, decreased, or stayed the same, which made it possible to interpret the trends.

Joinpoint regression was used to analyze trends in data over time and to identify periods of increasing or decreasing trend directions. We fit the joinpoint model to the data using the Joinpoint Trend Analysis Software V5.0. The standard error was used in the models and the significance level was set at 0.05. The annual percent change (APC) and the average annual percent change (AAPC) at each joinpoint were provided for every trend by the software to measure the rate of change in the variables over time.

### 2.2. Inclusion and Exclusion Criteria

CMS data included ixazomib, elotuzumab, and daratumumab prescriptions and reimbursements from Medicaid using the national drug codes (NDC) for all 50 states. Each data record included the NDC, drug name, year and quarter of Medicaid expenditure, number of pharmacy claims, number of units, and total pharmacy reimbursement amount, including the costs of the drug and its administration. We searched the database for each drug brand name and generic name. Using the unique NDC codes, we extracted all raw datasets. For each drug, annual prescription counts and reimbursement amounts were calculated by summing data across individual NDCs for each drug. During the study period, no generics were available for MM drugs, and the patent of the study drugs was still valid. The market share for utilization and reimbursement was also calculated for each drug. No exclusions were applied since this study included all data for the new MM medications, to capture the trends for each medication accurately.

Furthermore, the study estimated the market share of utilization and reimbursement for each MM. The market share was determined by dividing the utilization or spending of each drug by the total utilization or spending of all MM medications in each year. This estimate provided insights into the relative market share and impact of each medication within Medicaid.

## 3. Results

The overall Medicaid utilization of new MM medications rose from 1671 prescriptions in 2016 to 34,583 prescriptions in 2022 (1970% increase). Additionally, the overall Medicaid reimbursement for the new MM medications rose from over USD 9,250,000 in 2016 to over USD 214,449,000 in 2022, indicating a 2218% increase. The overall median amount of reimbursement per prescription for the three MM medications increased from USD 4892 in 2016 to USD 6014 in 2022 (a 23% increase) (Table 1).

### 3.1. MM Drug Utilization

#### 3.1.1. Ixazomib

**Figure 1:**
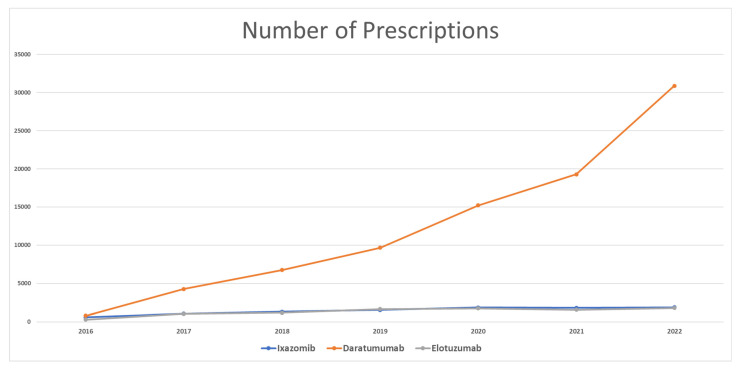
CMS utilization (number of prescriptions) for MM medications from 2016 to 2022.

In 2016, when ixazomib was approved by the FDA, its utilization was as low as 582 prescriptions. It increased every year, with a significant increase of 82% in 2017, which caused its utilization to reach 1059 prescriptions. Then, ixazomib continued to be issued in more prescriptions but with a slower pattern, with a 24% (1318 prescriptions), 16% (1530 prescriptions), and 21% (1854 prescriptions) increase in its utilization in 2018, 2019, and 2020, respectively. In 2021, ixazomib utilization decreased slightly by −3%, with 1806 prescriptions; then, it increased again in 2022 to 1890 prescriptions (5% increase from 2021). The total prescriptions for ixazomib from 2016 to 2022 amounted to 10,039 prescriptions. Utilization increased from 2016 to 2022 by 225% (Figure 1).

#### 3.1.2. Daratumumab

Once it appeared on the market, daratumumab showed remarkable utilization among MM medications. In 2016, the total prescriptions totaled 795 only. However, it reached 4279 prescriptions in 2017, with an increase in utilization by 438%. Daratumumab continued to be issued in more prescriptions, with an increase of 57%, 43%, and 57% in 2018, 2019, and 2020, respectively. In 2021, daratumumab increased in utilization by 27% only. Daratumumab utilization in 2022 increased to 30,895 prescriptions (60% increase from 2021). From 2016 to 2022, the total prescriptions for daratumumab amounted to 86,855. Daratumumab utilization increased from 2016 to 2022 by 3786%.

#### 3.1.3. Elotuzumab

Elotuzumab started with only 294 prescriptions in 2016, and then this increased to 1047 prescriptions in 2017 (256% increase). In the next three years, elotuzumab prescriptions continued to increase, by 11%, 43%, and 5% in 2018, 2019, and 2020, respectively. The utilization of elotuzumab decreased by 10% in 2021. In 2022, elotuzumab gained 1798 prescriptions, which resulted in an increase of 15% from 2021. Comparing 2016 and 2022, elotuzumab utilization increased by 512%.

### 3.2. MM Drug Reimbursement

#### 3.2.1. Ixazomib

**Figure 2:**
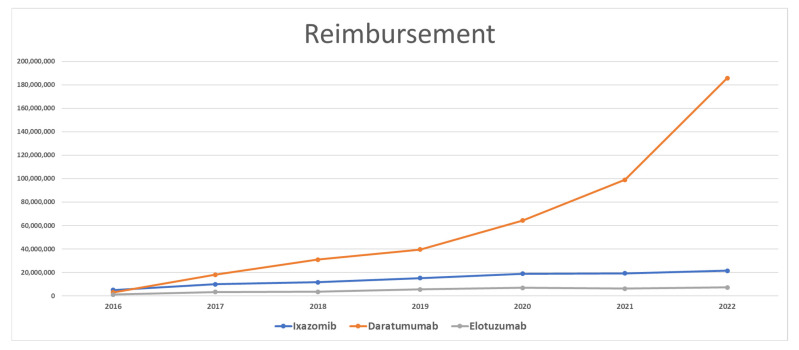
CMS spending (reimbursement) for MM medications from 2016 to 2022.

The reimbursement of ixazomib in 2016 amounted to over USD 5,096,000. Medicaid spending on Ixazomib almost doubled in 2017 and reached USD 10,008,905. Following 2017, spending continued increasing steadily until it reached USD 21,467,500 in 2022. Spending on ixazomib between 2016 and 2022 increased by over 321%. The total spending on ixazomib since 2016 is over USD 101,500,000 (Figure 2).

#### 3.2.2. Daratumumab

In the first year after entering the market, spending on daratumumab was as low as USD 2,937,741. However, in 2017, spending on daratumumab increased by 518% and reached over USD 18,000,000. Spending grew constantly by an average of 61% and reached USD 185,787,630 in 2022. Between 2016 and 2022, Medicaid’s spending on daratumumab increased by 3269%. The total spending on daratumumab since 2016 is over USD 440,569,000.

#### 3.2.3. Elotuzumab

Spending on elotuzumab was the lowest compared to its competitors. In 2016, the amount of elotuzumab reimbursement was USD 1,216,228. It increased by 181% and reached over USD 3,413,000 in 2017. Medicaid spending on elotuzumab slightly increased in 2018 by only 3%. In 2019 and 2020, elotuzumab was reimbursed in the amount of USD 5,473,792 (55% increase) and USD 6,941,713 (27% increase), respectively. In 2021, Medicaid spending on elotuzumab decreased by 10% and reached USD 6,254,204. In 2022, Medicaid spending on elotuzumab increased by 15% (USD 7,194,501) compared to 2021. The difference in spending between 2016 and 2021 increased by 492%. The total spending on elotuzumab since 2016 is over USD 34,025,000.

### 3.3. MM Drug Prices

#### 3.3.1. Ixazomib

**Figure 3:**
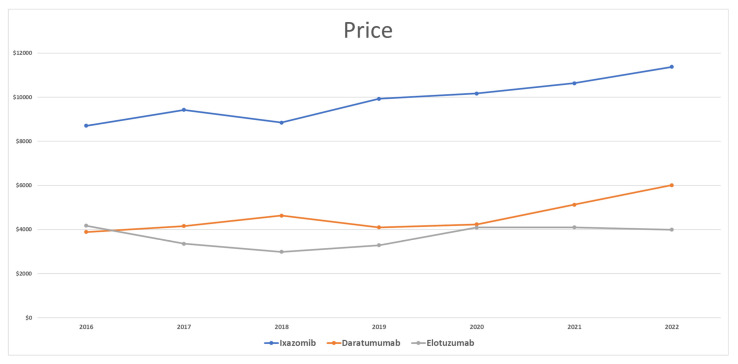
CMS price (reimbursement per prescription) for MM medications from 2016 to 2022.

Ixazomib started with an average price of USD 8706. Prices fluctuated, increasing by 8% in 2017 and decreasing by −6% in 2018. In 2019, ixazomib’s price increased and reached USD 9933 (12% increase). It continued to increase in the following years until it reached USD 11,380 per prescription in 2022. The difference in price between 2016 and 2022 was 31%. The average price for ixazomib was USD 9871 (Figure 3).

#### 3.3.2. Daratumumab

Daratumumab’s price in 2016 was as low as USD 3890 per prescription. Prices increased further in 2017 and 2018 by 7% and 11%, respectively. However, it decreased in 2019 by −11% and reached USD 4097 per prescription. In 2020, daratumumab’s prices slightly increased by only 3%, and then rose in 2021 to USD 5127 per prescription, with a 21% price increase. In 2022, daratumumab’s average price increased to USD 6014 (17% increase from 2021). The average price for daratumumab from 2016 to 2022 was USD 4593.

#### 3.3.3. Elotuzumab

In 2016, Medicaid paid an average price of USD 4172 for each elotuzumab prescription. In the following years, Medicaid paid less than this amount, by 20% (USD 3353) and 11% (USD 2991) in 2017 and 2018, respectively. In 2019 and 2020, prices for elotuzumab increased by 10% and 24%, respectively, and reached USD 4095 per prescription. No change in elotuzumab’s prices was noted in 2021, but it decreased by 3% in 2022 and reached USD 3993 per prescription. The average price for elotuzumab was USD 3714 for each prescription.

### 3.4. MM Drug Utilization Market Share

**Figure 4:**
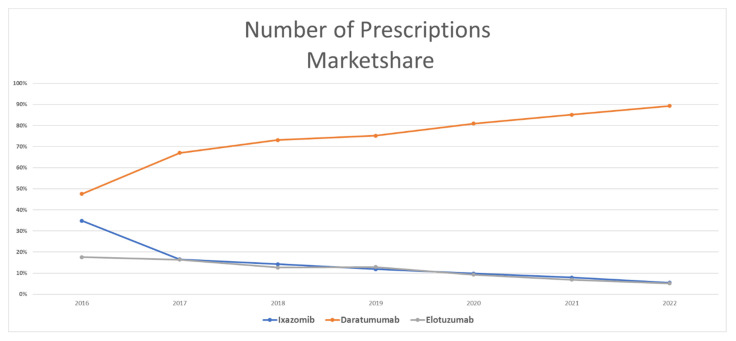
CMS utilization (number of prescriptions) market share for MM medications from 2016 to 2022.

Figure 4 shows the utilization market share for MM medications. Once they were approved by the FDA in late 2015, daratumumab captured an average of 45% of the market, followed by ixazomib with 37%, and elotuzumab with the remaining 18% of the market. In 2017, daratumumab took a greater market share than its competitors and occupied 66% of the market, while ixazomib and elotuzumab represented 17% each of the Medicaid utilization market. The same scenario was repeated in the following years with MM medications. Daratumumab dominated the market and increased its market share to 90% in 2022, while ixazomib and elotuzumab shared the remaining 10% of the market. The overall average market share utilization for MM medications since 2016 was 15%, 74%, and 12% for ixazomib, daratumumab, and elotuzumab, respectively.

### 3.5. MM Drug Reimbursement Market Shares

**Figure 5:**
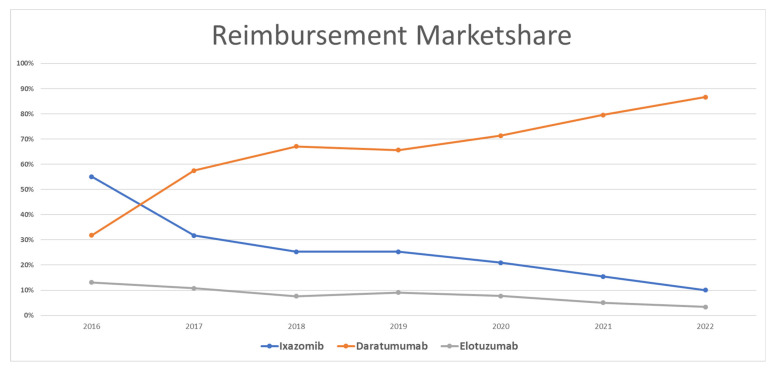
CMS spending (reimbursement) market share for MM medications from 2016 to 2022.

In terms of the reimbursement market share, ixazomib represented the largest Medicaid reimbursement in 2016, with 57%, while daratumumab and elotuzumab represented 30% and 13%, respectively. In the next year, daratumumab captured most of the market of MM medications, as it accounted for 56% of the market, while ixazomib represented 33% and elotuzumab only the remaining 11%. This market pattern continued for the rest of the study period, where daratumumab had the largest share of the market, with 87%, followed by ixazomib and elotuzumab with 10% and 3%, respectively. The overall average market share spending for MM medications since 2016 was 27%, 65%, and 8% for ixazomib, daratumumab, and elotuzumab, respectively (Figure 5).

### 3.6. Joinpoint Regression

**Figure 6:**
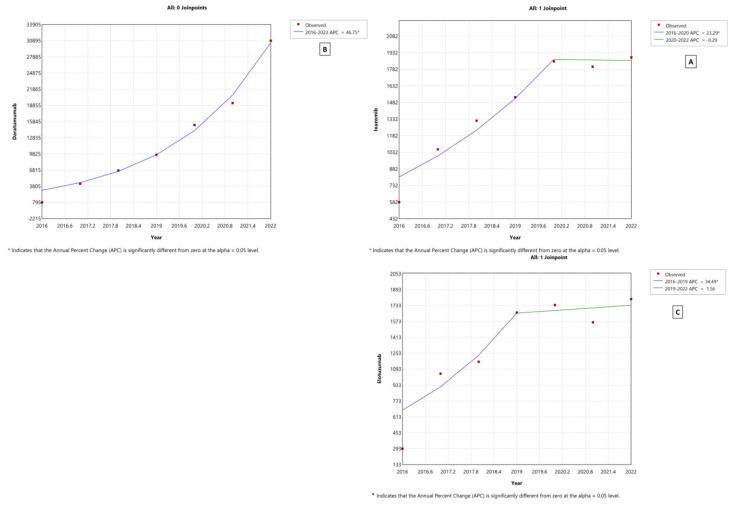
Joinpoint regression for MM medication utilization from 2016 to 2022 in CMS. (**A**) Ixazomib, (**B**) daratumumab, and (**C**) elotuzumab.

As shown in Figure 6, regarding MM medication utilization, the joinpoint regression indicated that there was a significant increase in the annual percent change (APC) from zero for daratumumab utilization from 2016 to 2022 (APC = 46.75, *p*-value < 0.05). For ixazomib, the APC showed a significant increase (APC = 23.29 *p*-value < 0.05) from 2016 to 2020. Then, the APC plateaued, with no significant difference for the rest of the study period. The APC for elotuzumab showed a significant increase between 2016 and 2019 (APC 34.49, *p*-value < 0.05), followed by a non-significant, slight increase in later years.

For reimbursement, the joinpoint model showed that daratumumab saw a non-significant increase between 2016 and 2020, followed by a significant APC increase from 2020 to 2022 (75.45, *p*-value < 0.05). It is pertinent to mention that the same model yielded a significant increase in the average annual percent change (AAPC) for daratumumab reimbursement from 2016 to 2022 (AAPC = 55, *p*-value < 0.05). Contrariwise, ixazomib and elotuzumab’s most significant APCs occurred from 2016 to 2020 (APC = 27.4 and 31.7, respectively, both *p*-values < 0.05). Both ixazomib and elotuzumab showed a significant AAPC (19.8 and 20.5, respectively, both *p*-values < 0.05) (Figure S1 in the Supplementary Materials).

In terms of price, the joinpoint regression showed a significant increase in daratumumab’s price from 2020 to 2022 (17.7, *p*-value < 0.05). Ixazomib had an APC increase (4.4, *p*-value < 0.05) for the whole study period, from 2016 to 2022. Elotuzumab’s APC decreased significantly between 2016 and 2018 (APC −12.4, *p*-value < 0.05), and then insignificantly increased in the rest of the study period (Figure S2 in the Supplementary Materials).

## 4. Discussion

This retrospective study utilized CMS claims data for three MM medications: ixazomib, elotuzumab, and daratumumab. The trend analysis revealed that Medicaid utilization and spending have increased enormously since 2016 for MM medications. Between 2016 and 2022, the overall MM medication utilization rose by over 1970%. This trend is reflective of the increased treatment rates of different types of cancer, particularly MM, due to improved survival rates [12,13]. Correspondingly, spending on these medications increased by over 2218% during the same period. This significant increase in Medicaid utilization and spending on MM medications is attribute to several factors. First, the improved survival rates due to advanced healthcare technology have led to longer treatment durations, resulting in the increased utilization of medications over time. Second, the introduction of newer, more expensive MM medications, such as daratumumab, has contributed to the rise in spending. These medications offer enhanced efficacy and improved patient outcomes, but they come at a higher cost. Additionally, the expansion of Medicaid and the implementation of the Affordable Care Act (ACA) have played a role in the increased utilization and spending on MM medications. These policy changes have improved access to healthcare services, including medications, for a larger population. As a result, more patients are seeking treatment for MM, leading to greater utilization and spending within the Medicaid program.

The trend of an increase in the utilization of, as well as the expenditure on, multiple myeloma (MM) medications can be ascribed to a number of considerable factors. These influencing aspects range from epidemiological changes to advancements in pharmacological interventions. Firstly, a salient factor underpinning this pattern is the noticeable rise in the incidence of MM within the United States as compared to prior decades. Recent statistical data indicate significant growth in the number of new cases, with an estimation that posits approximately 35,000 novel instances of MM in the US for the year 2021 alone, as per [5,14]. Therefore, MM accounts for around 10% of all hematological malignancies [15,16]. Secondly, the field of MM treatment has benefited from the advent of novel medications, whose remarkable efficacy has contributed significantly to the improvement of patient survival rates, as indicated in [12]. These new pharmacological interventions have brought forth a transformative change in prognosis, patient survival, and the management of MM. To substantiate this, evidence from clinical trials underscores the potency of these new medications. The results from a particular phase 3 trial provided compelling proof of the effectiveness of a combination therapy comprising daratumumab, lenalidomide, and dexamethasone. This therapeutic regimen was observed to significantly prolong not only the overall survival but also the progression-free survival among MM patients, serving as a testament to the promise that these innovative treatments hold in improving MM patient outcomes [13].

In a comparison of the three medications, daratumumab emerged as the medication with the highest utilization and reimbursement. This can be attributed to the superior efficacy of daratumumab compared with ixazomib and elotuzumab [17]. Daratumumab can be used for either newly diagnosed or relapsed MM patients. It is considered as a frontline drug in treating MM. It has been used as part of the triple regimen, daratumumab, lenalidomide, and dexamethasone, a regimen recognized as the current standard in managing MM [18,19]. Moreover, daratumumab can be used alone in relapsed MM patients, while ixazomib and elotuzumab are mainly used for relapsed MM patients [15]. The guideline that recommends using daratumumab as the first line of treatment for MM patients has had a significant impact on its utilization. This prescribing behavior is a sign of adherence to the guidelines, which is a good indication in managing patients with MM. Historically, adherence to clinical guidelines has been associated with improving recurrence-free survival, morbidity, and mortality [20,21].

The marked increase in daratumumab utilization in the past two years can be attributed to two factors. First, the FDA approved the new subcutaneous formula of daratumumab (Darzalex Faspro^®^) on 1 May 2020, which expanded its indication for newly diagnosed or relapsed/refractory multiple myeloma patients, providing healthcare providers with an additional treatment option in managing multiple myeloma [22]. Second, on 15 January 2021, the FDA approved Darzalex Faspro^®^ to treat patients with light-chain amyloidosis [23]. Due to its substantial efficacy in amyloidosis patients, Darzalex Faspro^®^ was incorporated into the new recommendations as a standard of care to manage amyloidosis patients [24]. These approvals have contributed to the increased utilization of daratumumab, as it is now recommended and prescribed for multiple myeloma and amyloidosis patients. Furthermore, the ease of use of the new subcutaneous daratumumab may have contributed to the increase in its utilization. Subcutaneous drugs are usually favored because they are less intrusive than intravenous (IV) medications, they are less expensive, and they have greater patient tolerability and thus higher patient adherence [25,26,27,28].

When analyzing the utilization of specific MM medications, ixazomib and elotuzumab had similar utilization over the years. However, there was a notable difference in reimbursement rates, primarily driven by the discrepancy in their pricing. Ixazomib, with its higher price, resulted in higher reimbursement compared to elotuzumab. The price of ixazomib was double that of daratumumab and elotuzumab. Interestingly, the price of elotuzumab remained relatively stable over the years, at around USD 4000 per prescription or even lower in some years. This is noteworthy considering that medication prices typically increase each year [29]. This consistency in elotuzumab’s pricing indicates a unique pricing strategy employed by the manufacturer. This strategy might involve pricing adjustments to maintain market competitiveness, negotiations with payers to secure favorable reimbursement rates, or specific agreements between the manufacturer and payers. The substantial increase in reimbursement reflects the growing utilization of these medications and the associated costs. These findings align with previous research that emphasized the significant financial burden imposed by MM medications on the healthcare system. It is important to note that multiple factors can influence these reimbursement trends, including changes in drug pricing, negotiation strategies, and healthcare policies. It is crucial to further investigate the underlying drivers of these reimbursement patterns to inform policy decisions and ensure fair and sustainable pricing for MM medications. Furthermore, the availability of generic alternatives and biosimilar compounds for MM medications can potentially introduce competition and reduce the prices, thereby enhancing affordability and access for Medicaid beneficiaries.

The overall increase in CMS spending and the utilization of medications is not solely limited to MM medications. Previous studies on different types of medications showed that CMS utilization and spending has increased significantly in the last few decades. This expansion was noticed in antidepressants, quinolone antibiotics, antirheumatics, anti-hypertensive, and asthma medications [30,31,32,33,34]. A report from the Centers for Medicare & Medicaid Services showed that Medicaid spending reached up to USD 734.0 billion in 2021, which is equal to 17% of the total national health expenditure [35]. Moreover, it is estimated that Medicaid spending will surpass USD 1 trillion by 2028 [35]. These findings highlight the substantial financial implications of these medications on the Medicaid program and align with previous studies that have reported escalating reimbursement costs for MM medications.

This pattern of increased utilization and expenditure is predicted to continue with daratumumab, since it has an active patent that prevents any competitor with biosimilar drugs from entering the market. The daratumumab brand drug Darzalex^®^ and the new subcutaneous formulation Darzalex Faspro^®^ are set to expire in the mid-2030s [36,37]. This means that the trend will continue unless new, efficacious drugs are approved by the FDA for MM. When biosimilar drugs become available, it is likely that the use of daratumumab will decrease. Biosimilar drugs could make the medication more affordable and accessible to patients with different types of insurance and those who need it.

The study also found that policy and disease management guidelines can have a major impact on the use and costs of MM medications. For instance, the CMS has implemented several policies leading to increased daratumumab use. The coverage of daratumumab in an outpatient setting, reimbursement for daratumumab in combination with other MM drugs, and the expansion of daratumumab access through the Medicare Part D Prescription Drug Plan are all examples of such policies. The findings of this study have significant implications for patients with multiple myeloma (MM), healthcare providers, and policymakers. Patients with MM need to be aware of the rising costs of MM treatment and the options available to them. Healthcare providers need to be aware of the most recent treatment guidelines and the impact of policies on the use and spending of MM medications. Policymakers need to consider the impact of their policies on the use and spending of MM medications.

The rising use and expenditure of MM drugs present a serious challenge to the healthcare sector. Balancing the need for cost control while ensuring that patients have access to the necessary care is a complex task. To address these challenges, it is critical to develop strategies that control expenditures while ensuring that patients have access to the care that they require. One approach is the implementation of evidence-based treatment guidelines and formulary management practices. By promoting the use of cost-effective medications and ensuring appropriate utilization, healthcare providers can help to control spending while maintaining high-quality care for MM patients. Additionally, exploring alternative payment models and negotiating drug prices can contribute to cost containment efforts. These challenges also can be addressed by including comparative effectiveness research (CER) to evaluate the relative benefits and risks of different treatment options. Furthermore, adopting patient-centered healthcare delivery models that prioritize individual preferences and values, as well as designing oncology drug benefit programs, can optimize both clinical outcomes and cost-effectiveness [38]. Incorporating patient-reported outcomes (PROs) into the care of patients with MM shows promise in improving the quality and cost-effectiveness of treatment [39]. PROs provide valuable insights into patient experiences and outcomes, enabling healthcare providers to tailor treatment plans and improve overall patient care.

While this study offers valuable insights into the utilization, reimbursement, and pricing trends of multiple myeloma medications within the Medicaid program, there are certain limitations that should be considered. Firstly, the generalizability of the findings could be limited, as the study focused exclusively on the Medicaid program. Thus, the utilization, reimbursement, and pricing patterns observed in Medicaid may not be representative of other populations and cannot be generalized to other types of public or private insurance. Additionally, the study relied on administrative claims data, which might be subject to inherent limitations in accuracy and completeness. However, CMS data represent more than 34% of the US population, accounting for more than 100 million enrollees [40].

Moreover, the study primarily examined utilization, reimbursement, and pricing data, without considering patient demographics, efficacy, safety, or clinical outcomes. This limitation potentially hinders a comprehensive understanding of the impact of these medications on patient care and health outcomes. Moreover, a significant limitation is that the study used retrospective pharmacy claims data. Such data might be subject to missing or incomplete information, coding errors, or other inaccuracies that could affect the overall results. These limitations arise from the administrative nature of the data, which are collected primarily for reimbursement rather than research purposes. Moreover, the inability to verify the accuracy of the pharmacy claims data, such as a specific state’s data or patients’ specific characteristics, is an inherent challenge in such analyses. Additionally, given that our study was observational in nature, it inherently lacked the ability to infer causation. We could only determine associations between variables, and we cannot conclude that one variable caused a change in another. Another limitation is the potential for confounding factors that were not accounted for in our study. Although we examined utilization, reimbursement, and pricing data, we did not have access to patient-specific clinical data or comorbid conditions, which might influence medication utilization and spending patterns. Lastly, our study did not account for the potential influence of marketing or promotional activities by pharmaceutical companies, which can significantly affect medication utilization trends. Despite these limitations, this study serves to enrich our understanding of the utilization, reimbursement, and pricing trends of MM medications within the Medicaid program. Moreover, the findings of this study can assist in improving patient care by identifying areas where cost savings can be achieved without sacrificing quality. Analyzing medication utilization patterns allows healthcare providers to identify opportunities to optimize treatment regimens, reduce medication waste, and enhance patient adherence. This can lead to better treatment outcomes, improved patient satisfaction, and the efficient use of healthcare resources. The study’s findings can also contribute to ongoing efforts to enhance medication’s affordability and accessibility for Medicaid beneficiaries. It is worth emphasizing that further research is necessary to validate and expand upon these findings, including a broader range of MM medications and incorporating additional outcome measures to provide a more comprehensive assessment of the economic implications of MM treatment.

## 5. Conclusions

The findings of this study demonstrate that the overall CMS utilization and spending on MM medications have increased remarkably since 2016. Daratumumab showed the highest utilization, spending, and market share compared to ixazomib and elotuzumab. Ixazomib had the highest price compared to other MM medications. Evidently, policy and disease management guidelines have a significant impact on the utilization and spending of certain medications. The findings of this study have significant implications for the management of MM. Policymakers and healthcare providers need to be cognizant of the factors driving the utilization and spending of MM medications, including policy and disease management guidelines. By understanding these factors, they can develop strategies to ensure that patients have access to the most effective treatments at an affordable cost.

## Figures and Tables

**Table 1:** This table summarizes the primary parameters of this study, which included CMS utilization (number of prescriptions), spending (reimbursement in US dollars), price (reimbursement per prescription in US dollars), and market share (%) for MM medications from 2016 to 2022. Each year has five rows (four quarters and the total/average).

Year	Quarter	Number of Prescriptions (Utilization)			Total Spending (Reimbursement) ($)			Price (Reimbursement Per Prescription) ($)			Market Share (%)					
		Ixazomib	Daratumumab	Elotuzumab	Ixazomib	Daratumumab	Elotuzumab	Ixazomib	Daratumumab	Elotuzumab	Number of Prescriptions (Utilization)			Total Spending (Reimbursement)		
											Ixazomib	Daratumumab	Elotuzumab	Ixazomib	Daratumumab	Elotuzumab
**2016**	Q1	49	59	26	418,974	184,150	85,397	8550	3121	3285	37	44	19	61	27	12
	Q2	124	103	56	1,075,014	548,096	249,870	8669	5321	4462	44	36	20	57	29	13
	Q3	188	248	73	1,640,289	966,903	400,191	8725	3899	5482	37	49	14	55	32	13
	Q4	221	385	139	1,962,145	1,238,592	480,770	8878	3217	3459	30	52	19	53	34	13
	Total/Average	582	795	294	5,096,423	2,937,741	1,216,228	8706	3890	4172	37	45	18	57	30	13
**2017**	Q1	213	588	172	1,921,693	2,124,497	711,852	9022	3613	4139	22	60	18	40	45	15
	Q2	258	1105	282	2,459,439	4,685,435	993,303	9533	4240	3522	16	67	17	30	58	12
	Q3	272	1091	325	2,609,437	4,800,696	950,493	9594	4400	2925	16	65	19	31	57	11
	Q4	316	1495	268	3,018,335	6,537,390	757,884	9552	4373	2828	15	72	13	29	63	7
	Total/Average	1059	4279	1047	10,008,905	18,148,017	3,413,532	9425	4157	3353	17	66	17	33	56	11
**2018**	Q1	313	1514	236	2,845,100	8,413,147	576,247	9090	5557	2442	15	73	11	24	71	5
	Q2	334	1618	332	2,710,233	7,070,271	1,050,124	8114	4370	3163	15	71	15	25	65	10
	Q3	331	1665	298	2,707,603	7,519,141	922,973	8180	4516	3097	14	73	13	24	67	8
	Q4	340	1942	301	3,401,326	7,906,849	982,065	10,004	4071	3263	13	75	12	28	64	8
	Total/Average	1318	6739	1167	11,664,261	30,909,408	3,531,409	8847	4629	2991	14	73	13	25	67	8
**2019**	Q1	343	2287	362	3,316,103	10,286,895	1,241,526	9668	4498	3430	11	76	12	22	69	8
	Q2	406	2526	418	4,034,498	9,921,096	1,465,808	9937	3928	3507	12	75	12	26	64	10
	Q3	415	2617	475	4,183,645	10,570,097	1,598,221	10,081	4039	3365	12	75	14	26	65	10
	Q4	366	2228	408	3,677,176	8,737,377	1,168,238	10,047	3922	2863	12	74	14	27	64	9
	Total/Average	1530	9658	1663	15,211,421	39,515,466	5,473,792	9933	4097	3291	12	75	13	25	66	9
**2020**	Q1	400	4048	551	4,022,046	16,046,282	1,669,349	10,055	3964	3030	8	81	11	19	74	8
	Q2	499	3839	449	5,030,400	15,736,047	1,943,357	10,081	4099	4328	10	80	9	22	69	9
	Q3	476	3639	371	4,876,649	15,865,742	1,613,262	10,245	4360	4348	11	81	8	22	71	7
	Q4	479	3676	367	4,934,713	16,638,493	1,715,746	10,302	4526	4675	11	81	8	21	71	7
	Total/Average	1854	15,202	1738	18,863,808	64,286,564	6,941,713	10,171	4237	4095	10	81	9	21	71	8
**2021**	Q1	485	4341	328	5,139,634	21,125,781	1,724,861	10,597	4867	5259	9	84	6	18	75	6
	Q2	464	4631	364	5,014,912	24,167,953	1,451,278	10,808	5219	3987	8	85	7	16	79	5
	Q3	456	5366	506	4,828,613	27,089,448	1,658,160	10,589	5048	3277	7	85	8	14	81	5
	Q4	401	4949	366	4,225,339	26,601,581	1,419,905	10,537	5375	3880	7	87	6	13	82	4
	Total/Average	1806	19,287	1564	19,208,499	98,984,763	6,254,204	10,633	5127	4101	8	85	7	16	79	5
**2022**	Q1	640	10,259	701	7,236,890	60,599,589	2,754,584	11,308	5907	3930	6	88	6	10	86	4
	Q2	644	9989	558	7,296,106	61,131,335	2,278,834	11,329	6120	4084	6	89	5	10	86	3
	Q3	333	6152	329	3,814,904	37,095,479	1,355,151	11,456	6030	4119	5	90	5	9	88	3
	Q4	273	4495	210	3,119,600	26,961,227	805,931	11,427	5998	3838	5	90	4	10	87	3
	Total/Average	1890	30,895	1798	21,467,500	185,787,630	7,194,501	11,380	6014	3993	5	90	5	10	87	3
**Total/Average**		**10,039**	**86,855**	**9271**	**101,520,817**	**440,569,590**	**34,025,380**	**9871**	**4593**	**3714**	**15**	**74**	**12**	**27**	**65**	**8**

## Data Availability

CMS data are publicly available from the Centers for Medicare & Medicaid Services at https://www.medicaid.gov/medicaid/prescription-drugs/state-drug-utilization-data/index.html. accessed on 1 April 2023.

## Supplementary Materials

The following supporting information can be downloaded at: https://www.mdpi.com/article/10.3390/healthcare11162265/s1.
