# Supplementary material for: Drug Expenditure, Price, and Utilization in US Medicaid: A Trend Analysis for New Multiple Myeloma Medications from 2016 to 2022
Source: Healthcare (Basel). 2023 Aug 11;11(16):2265. doi: 10.3390/healthcare11162265 (PMC10454759; doi:10.3390/healthcare11162265)
Supplement: Supplementary file 1 [file healthcare-11-02265-s001.zip › healthcare-2484491-supplementary.pdf]

Appendix

Year	Ixazomib	Daratumumab	Elotuzumab
**2016**	582	795	294
**2017**	1059	4279	1047
**2018**	1318	6739	1167
**2019**	1530	9658	1663
**2020**	1854	15202	1738
**2021**	1806	19287	1564
**2022**	1890	30895	1798
**Total**	**10039**	**86855**	**9271**
**Mean**	**1434**	**12408**	**1324**
**SD**	**485**	**10308**	**537**

Table S1: Descriptive table for MM medication utilization in CMS from 2016 to 2022.

Year	Ixazomib ($)	Daratumumab ($)	Elotuzumab ($)
**2016**	5,096,423	2,937,741	1,216,228
**2017**	10,008,905	18,148,017	3,413,532
**2018**	11,664,261	30,909,408	3,531,409
**2019**	15,211,421	39,515,466	5,473,792
**2020**	18,863,808	64,286,564	6,941,713
**2021**	19,208,499	98,984,763	6,254,204
**2022**	21,467,500	185,787,630	7,194,501
**Total**	**101,520,817**	**440,569,590**	**34,025,380**
**Mean**	**4,782,666**	**45,783,834**	**1,834,610**
**SD**	**5872300.9**	**62681521.5**	**2207504.8**

Table S2: Descriptive table for MM medication reimbursement in CMS from 2016 to 2022.

Year	Ixazomib ($)	Daratumumab ($)	Elotuzumab ($)
**2016**	8,706	3,890	4,172
**2017**	9,425	4,157	3,353
**2018**	8,847	4,629	2,991
**2019**	9,933	4,097	3,291
**2020**	10,171	4,237	4,095
**2021**	10,633	5,127	4,101
**2022**	11,380	6,014	3,993
**Mean**	**9,871**	**4,593**	**3,714**
**SD**	**962.3222**	**748.6815**	**485.3589**

Table S3: Descriptive table for MM medication price in CMS from 2016 to 2022.

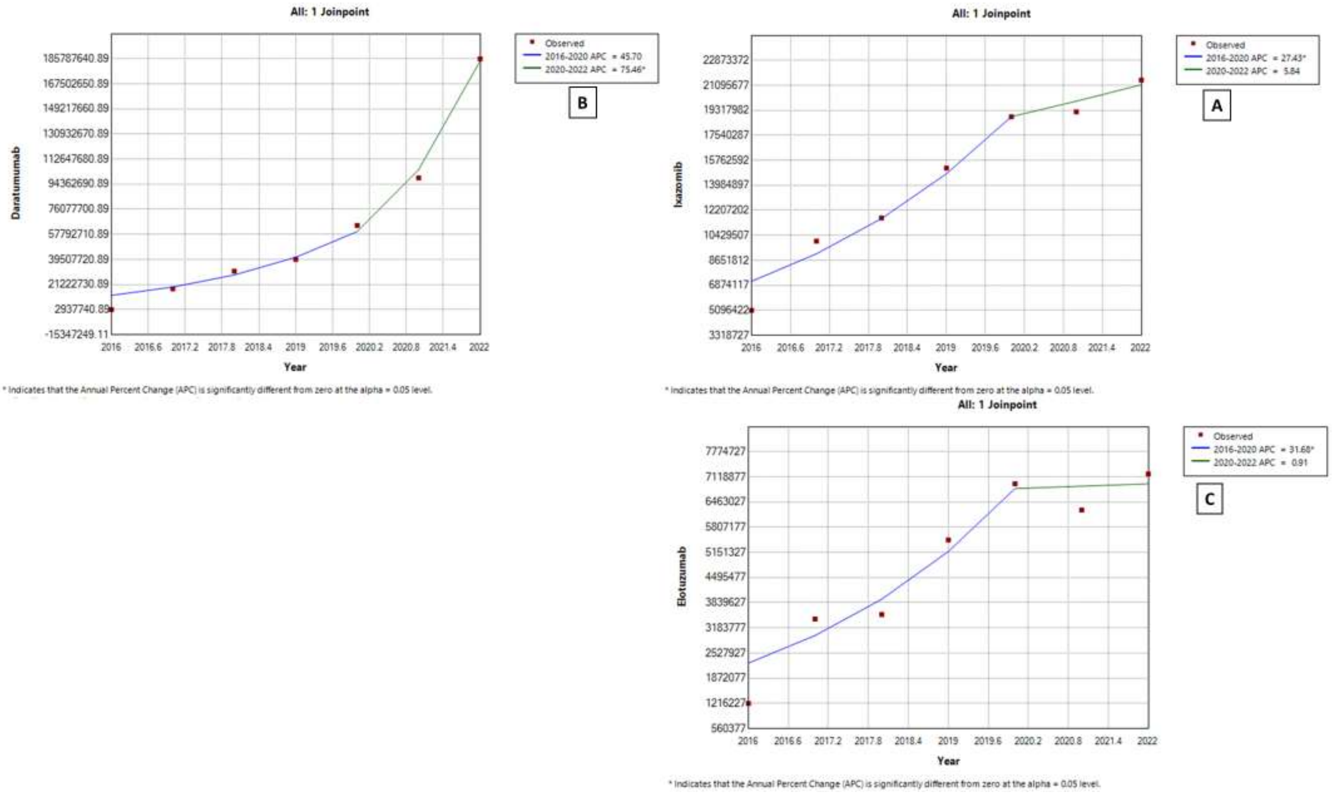

Figure S1: Joinpoint regression for MM medication reimbursement from 2016 to 2022 in CMS. Figure A for ixazomib, B for daratumumab, and C for elotuzumab.

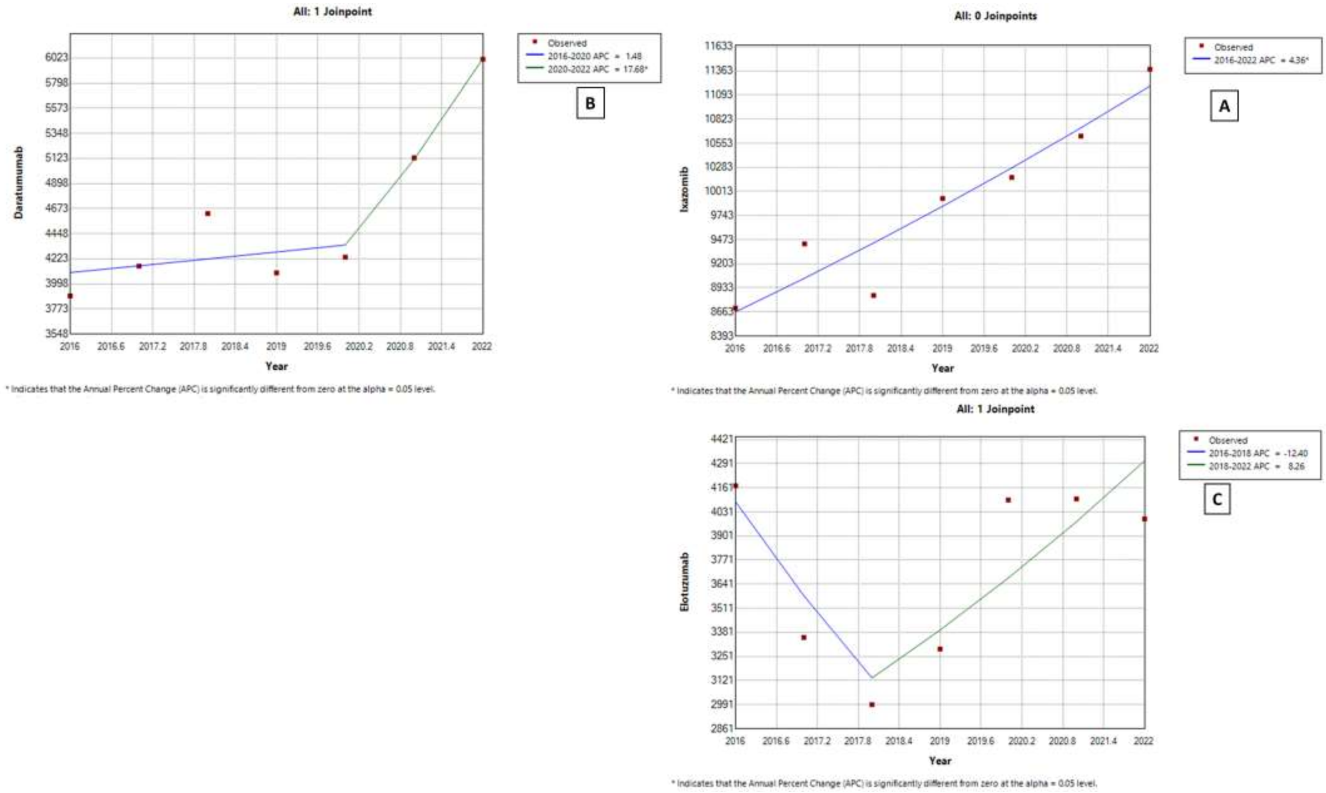

Figure S2: Joinpoint regression for MM medication price from 2016 to 2022 in CMS. Figure A for ixazomib, B for daratumumab, and C for elotuzumab.

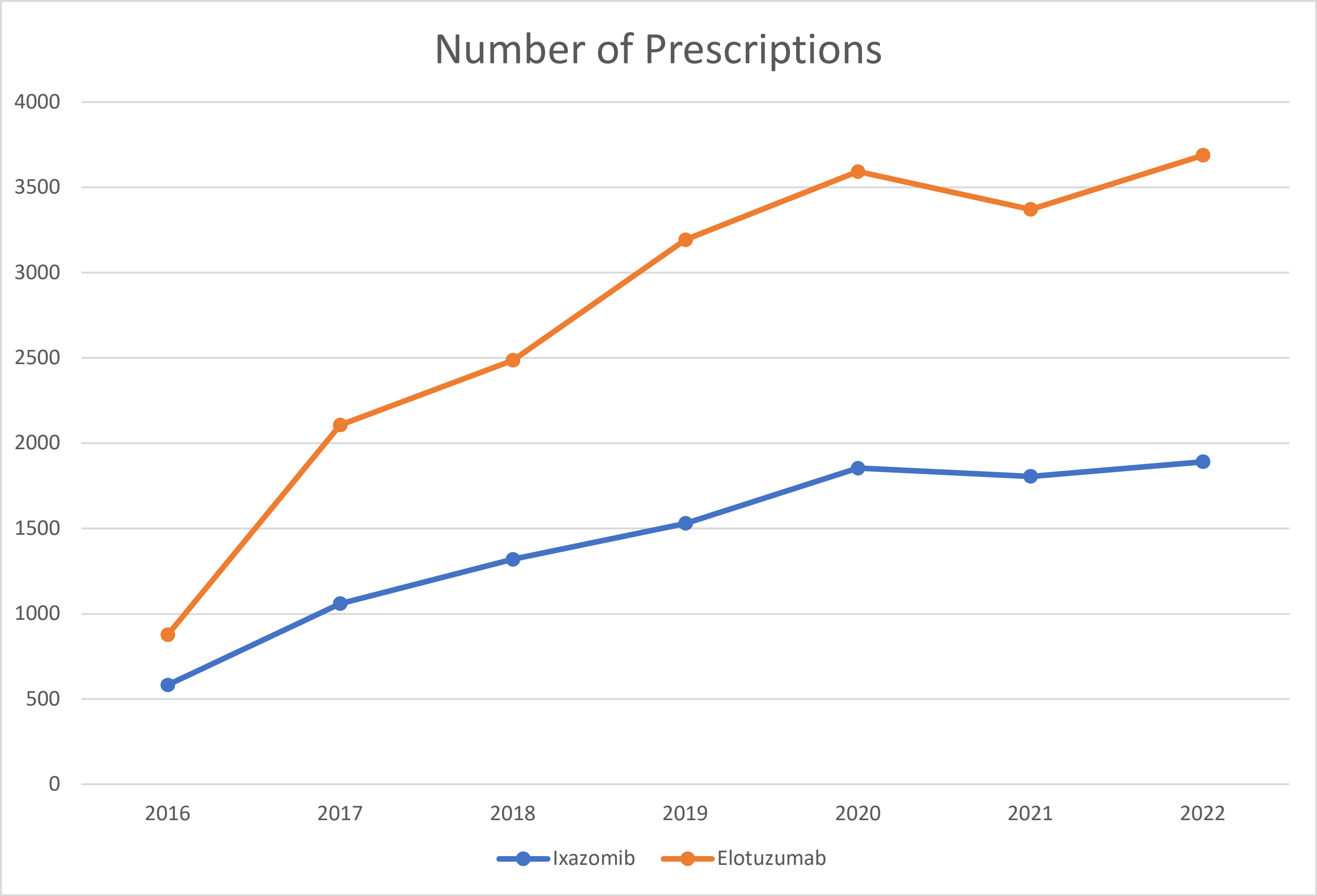

Figure S3: CMS utilization for MM medications from 2016 to 2022
